# Cherry Anthocyanins Regulate NAFLD by Promoting Autophagy Pathway

**DOI:** 10.1155/2019/4825949

**Published:** 2019-02-25

**Authors:** Qiang Chu, Shuang Zhang, Meng Chen, Wen Han, Ruoyi Jia, Wen Chen, Xiaodong Zheng

**Affiliations:** ^1^Department of Food Science and Nutrition, Zhejiang University, Hangzhou 310058, China; ^2^Zhejiang Key Laboratory for Agro-food Processing, Zhejiang University, Hangzhou 310058, China; ^3^Fuli Institute of Food Science, Zhejiang University, Hangzhou 310058, China; ^4^College of Environmental and Resource Science, Zhejiang University, Hangzhou 310058, China

## Abstract

Nonalcoholic fatty liver disease (NAFLD) is a common chronic disease that threatens human health, and present therapies remain limited due to the lack of effective drugs. Lipid metabolic disturbance and oxidative stress have strong links to the development of NAFLD, while autophagy was generally accepted as a key regulatory mechanism on these steps. Our previous studies indicated that cherry anthocyanins (CACN) protected against high fat diet-induced obesity and NALFD in C57BL/6 mice, while the underlying molecule mechanism is still unclear. Thus, in this study, we show that CACN protect against oleic acid- (OA-) induced oxidative stress and attenuate lipid droplet accumulation in NAFLD cell models. According to the results of a transmission electron microscope (TEM), western blot, immunofluorescence (IF), and adenovirus transfection (Ad-mCherry-GFP-LC3B), autophagy is in accordance with the lipid-lowering effect induced by CACN. Further studies illustrate that CACN may activate autophagy via mTOR pathways. In addition, an autophagy inhibitor, 3-methyladenine (3-MA), was applied and the result suggested that autophagy indeed participates in the lipid clearance process in OA-induced lipid accumulation. All these results indicate that the positive effects of CACN on OA-induced hepatic lipid accumulation are mediated via activating autophagy, showing a potential target for the therapeutic strategy of NAFLD.

## 1. Introduction

Nonalcoholic fatty liver disease, characterized by excess triglyceride (TG) and continuous oxidative stress in liver cells, is commonly associated with metabolic syndrome and cardiovascular diseases and emerges as the early form of steatosis before progressing to chronic liver disease [1–3]. About 30–40% adults of the general population are considered to have superfluous liver fat accumulation [4]. A newly published study indicated that in North America, Europe, and Asia, over 30% of the people suffered from obesity, more than 50% of them have type 2 diabetes, and even nearly 100% obese patients are accompanied with NAFLD [5]. The nosogenesis of NAFLD is complex, and the “two-hit” hypothesis is the most well-known theory regarding the pathogenesis of NAFLD [6]. The overaccumulation of intracellular lipids is the “first hit,” which will lead to the “second hit” factors, such as oxidative stress, inflammation, and mitochondrial dysfunction, resulting in the hepatocyte injury, fibrosis, and apoptosis [7]. These pathologic changes may perturb the activation and execution of autophagy in different cells. Researchers illustrated that autophagy could block NAFLD development by digesting the intracellular hepatocyte lipid droplets and the specific autophagy in modulating intracellular lipid accumulation is called lipophagy [8].

The inhibition of autophagy by the knockdown of the autophagy-related genes (Atgs) or pharmacological treatment with 3-MA in cultured hepatocytes can obviously increase intracellular triglyceride (TG) storage, lipid droplet number, and size, in response to lipid challenge [9]. Another study reported that caffeine activates hepatic lipid mechanism and enhances hepatic lipid droplets clearance by the autophagy-lysosome pathway [10], which confirms that autophagy is a new underlying therapeutic target for NAFLD.

Although several drugs, including volixibat and aramchol, are demonstrated to be effective in alleviating NAFLD, there is still no U.S. Food and Drug Administration- (FDA-) approved drug for the treatment of it, despite it becoming the most common liver disease around the world [11]. However, due to the potentially toxic or side effects of some anti-NAFLD drugs, such as orlistat and sibutramine [12], searching natural phytochemical compounds provides an efficient approach to prevent NAFLD. Anthocyanins (ACNs) are widely found in a lot of berry fruits, such as cherry, mulberry, blueberry, strawberry, cranberry, and waxberry, which have become an indispensable part of human diet [13, 14]. It is generally accepted that ACNs possess multiple biological activities including antioxidation, anti-inflammation, antidiabetes, obesity control, cardiovascular disease prevention, and visual and brain function enhancement [15–22]. Sweet cherry (*Prunusauiun* L.) is a nutritious food with relatively low caloric content and large amounts of important bioactive food factors such as cyanidin-3-glucoside and cyanidine-3-rutinoside [23]. Our previous reports suggested that dietary purified sweet cherry anthocyanins could markedly decrease high-fat diet-induced obesity, insulin resistance, and hepatic steatosis in C57BL/6 mice [24, 25]. However, the underlying molecular mechanisms of CACN on hepatic steatosis were not fully illuminated. This study is aimed at purifying CACN from sweet cherry and evaluating the potential molecular mechanism of CACN on OA-induced lipid accumulation. Moreover, we explored the potential role of autophagy in the beneficial effects of CACN on hepatic lipid accumulation.

## 2. Material and Methods

### 2.1. Material and Reagents

Fresh sweet cherry was purchased from a fruit market in Hangzhou. 2-NBDG was obtained from ApexBio. HepG2 cells and LO2 cells were obtained from Type Culture Collection of the Chinese Academy of Sciences (Shanghai, China). 2,7-Dichlorodihydrofluorescein diacetate (DCF-DA), dihydroethidium (DHE), naphthalene-2,3-dicarboxaldehyde (NDA), rhodamine 123 (RH123), and 3-methyladenine (3-MA) were obtained from the Lifetech (Shanghai, China). DAPG (D676) autophagy detection probe was purchased from Dojindo (Shanghai, China). Control-siRNA and Atg5-siRNA, containing three target sequences, were obtained from RiboBio (Guangzhou, China). Primary and secondary antibodies for western blot analysis were obtained from Abcam (Shanghai, China). Chloroquine (CQ), LC3 (SAB1305639), Atg7 (HPA007639), Beclin1 (SAB4100184), and p62 (P0067) primary antibodies for immunofluorescence analysis were obtained from Sigma-Aldrich (Shanghai, China). Adenovirus expressing mCherry-GFP-LC3B fusion protein (Ad-mCherry-GFP-LC3B), LysoTracker Green, Nile Red, WB/IP lysis buffer, and ECL Western blotting system were purchased from Beyotime Biotechnology (Jiangsu, China). BCA Protein Assay Kit, TG Assay Kit, and TC Assay were purchased from Jiancheng Biotechnology (Nanjing, China). Other reagents were of reagent grade and obtained from Aladdin (Shanghai, China).

### 2.2. Preparation and Determination of Sweet Cherry Anthocyanins

The method for the isolation and identification of CACN was according to our previous study with slight modifications [25]. Briefly, sweet cherry was extracted with 95% ethanol containing 1% HCl for 10 h in a shaking water bath at 4°C. Filtered fluid was evaporated and centrifuged, loaded onto an AB-8 macroporous resin column, and then orderly eluted with 1% formic acid in 90% ethanol for further purification. Finally, purified CACN was got by lyophilization and stored at −80°C for further analysis. Composition and content of anthocyanins in MAE were determined by an HPLC instrument (Thermo UltiMate 3000). The total contents of polyphenols and flavonoids were determined by the Folin-Ciocalteu and the aluminum chloride colorimetric methods using gallic acid (GA) and rutin (R) as standards, respectively.

### 2.3. Cell Culture and Treatments

HepG2 and LO2 cells were maintained in Dulbecco's modified Eagle's medium (DMEM) supplemented with 10% heat-inactivated fetal bovine serum, penicillin (100 units/mL), and streptomycin (100 *μ*g/mL) at 37°C in 5% CO_2_ atmosphere. After reaching 80% confluence, HepG2 and LO2 cells were seeded into various cell culture plates for 24 h. After being attached, cells were cotreated with different concentrations of CACN and 0.5 mM OA for another 24 h [10]. Cells solely incubated with 0.5 mM OA were regarded as the model group.

### 2.4. Cell Viability Assay

After reaching 80% confluence, cells were digested and seeded into a 96-well plate. The following day, cells were treated with different concentrations of CACN for 24 h. At the end of the treatment, the medium was carefully discarded and fresh medium containing 0.5 mg/mL methyl-thiazolyl-tetrazolium (MTT) was subsequently added to each well followed by additional 4 h incubation at 37°C. Finally, the MTT formazan precipitate was dissolved in 150 *μ*L DMSO and the absorbance was measured at 570 nm using a microplate spectrophotometer [26].

### 2.5. Fluorescent Staining

After treatments, LO2 cells were treated with 5 *μ*M DCF-DA, 5 *μ*M DHE, 40 *μ*M NDA, 10 *μ*M RH123, 50 nM LysoTracker Green, 10 *μ*g/mL Nile Red, and 0.1 *μ*M DAPG in serum-free DMEM for 30 min at 37°C to determine intracellular ROS production, O_2_^−^ generation, glutathione (GSH) depletion, mitochondrial membrane potential, lysosome, oil droplet, and autophagosome. Subsequently, the medium was replaced by fresh medium without phenol red and immediately detected by a fluorescence microscope at the same exposure time [27].

### 2.6. Oil Red O Staining

After treatments, HepG2 and LO2 cells were washed three times with cold phosphate-buffered saline (PBS) and fixed in 4% paraformaldehyde for 15 min. Then, the cells were washed twice with PBS and stained with 0.5% oil red O for 30 min. Subsequently, the cells were further washed using distilled water to remove the unbound oil red O for the optical microscopy observation. Finally, to quantitate the lipid content, 150 *μ*L of isopropanol was added to each well and plates were read at 510 nm using a microplate spectrophotometer after shaking at room temperature for 15 min.

### 2.7. Intracellular Triglyceride (TG) and Total Cholesterol (TC) Measurements

At the end of treatment, the cells were washed with cold PBS and then lysed with cell lysis buffer containing 1% Triton X-100. The concentrations of intracellular TG and TC were measured using commercially available assay kits according to the manufacturer's instructions. And the total protein in the cell lysate was measured by a BCA Protein Assay Kit.

### 2.8. Transmission Electron Microscope Analysis

HepG2 and LO2 cells were digested and centrifuged at 800 rpm for 5 min. Subsequently, the cell pellet was collected, double fixated, dehydrated, infiltrated, embedded, ultrathin sectioned, stained, and observed in a Hitachi Model H-7650 TEM [28].

### 2.9. Immunofluorescence Analysis

HepG2 and LO2 cells were grown on glass coverslips and treated as described previously. Then, cells were fixed with 4% paraformaldehyde, permeabilized with 0.2% triton X-100, and blocked with PBS containing 5% goat serum albumin. After incubation with primary antibodies at 4°C overnight, cells were washed five times with PBS, incubated with DAPI and FITC-conjugated second antibody in blocking buffer for 1.5 h at 37°C, and observed by a fluorescence microscope.

### 2.10. Adenovirus Transfection (Ad-mCherry-GFP-LC3B)

HepG2 and LO2 cells were seeded into a 24-well plate and transfected with Ad-mCherry-GFP-LC3B at 30 multiplicities of infection (MOI) for 24 h after being attached. Then, the cells were cotreated with different concentrations of 0.5 mM OA, CACN, or 3-MA for another 24 h. After treatments, the mCherry-GFP-LC3B fusion protein was visualized with the fluorescence microscope.

### 2.11. Western Blot Analysis

Western blot was performed as described previously with slight modifications [17]. Briefly, HepG2 and LO2 cells were lysed in WB/IP lysis buffer at 4°C after treatments, and then, the lysates were centrifuged at 12,000 g for 10 min, and the protein contents were measured with a BCA Kit. Subsequently, the same amounts of total protein were separated by 12% SDS-PAGE, transferred to PVDF membranes, blocked, and blotted with different primary antibodies. Immunoreactive bands were then visualized using proper peroxidase-conjugated secondary antibodies, and the protein bands were quantified using ImageJ software [29].

### 2.12. Statistical Analysis

All data are expressed as the mean ± SD from three measurements at least. The statistical analysis was determined through one-way analysis of variance followed by ANOVA tests using the SPSS 20.0. And *p* < 0.05 was considered to be statistically significant.

## 3. Results

### 3.1. Ingredients of CACN

**Figure 1:**
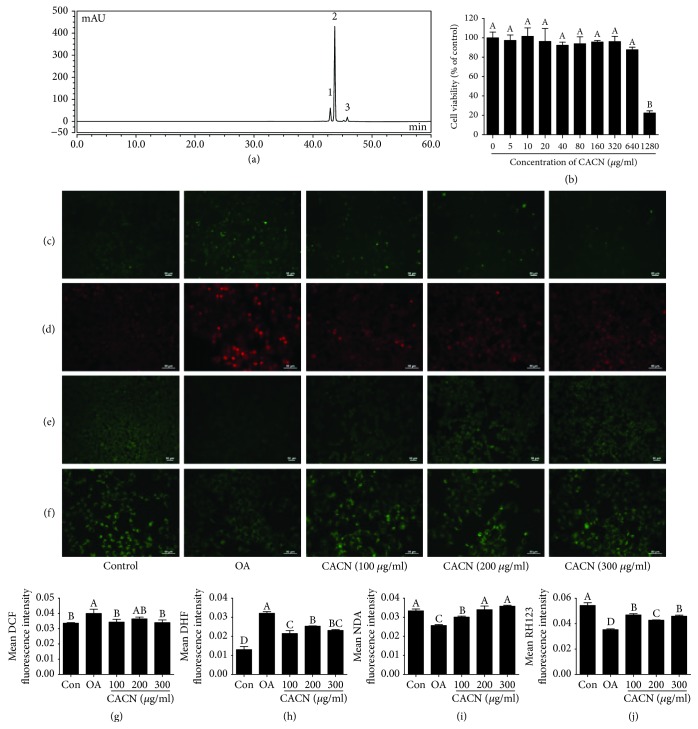
HPLC elution profiles of CACN and effects of CACN on OA-induced oxidative stress in LO2 cells. (a) HPLC profile of CACN. (b) Cell viability assay. (c) DCF staining for intracellular ROS. (d) DHE staining for intracellular O_2_^−^. (e) NDA staining for intracellular GSH. (f) RH123 staining for mitochondrial membrane potential (MMP). (g), (h), (i), and (j) represent the quantitative results of (c), (d), (e), and (f), respectively.

The compositions and contents of the purified CACN were measured by HPLC. Results showed that the retention times for cyanidin-3-glucoside (C-3-G), cyanidin-3-rutinoside (C-3-R), and pelargonidin-3-glucoside(P-3-G) were 42.9, 43.6, and 45.8 min, respectively (Figure 1(a)), which correspond with those of previous studies. And 1 g of the CACN contained 123.70 ± 3.22 mg C-3-G, 622.88 ± 8.27 mg C-3-R, and 62.66 ± 1.17 mg P-3-G. The total phenolic and flavonoid contents of 1 g CACN, expressed as the gallic acid and rutin equivalent, were 770.99 ± 6.45 mg GA and 870.08 ± 8.53 mg R, respectively. And the percentage of ACNs accounted for >80%, indicating that ACNs play a major role in the biological activity of the sweet cherry extract.

### 3.2. CACN Prevented OA-Induced Oxidative Stress in LO2 Cells

Different concentrations of CACN were incubated with LO2 cells to detect the cytotoxicity. As shown in Figure 1(b), incubation of LO2 cells with CACN from 5 to 640 *μ*g/mL did not markedly reduce cell viability, suggesting CACN at this range of concentrations exerted no measurable cell toxicity, which could be used for further research. Previous studies have reported that OA-induced lipid deposition led to oxidative stress, inflammation, and mitochondrial dysfunction [30]. Thus, we investigated the protective effects of CACN on OA-induced oxidative stress and mitochondrial dysfunction. Firstly, DCF and DHE fluorescent probes were used to measure the intracellular ROS and O_2_^−^ levels. As expected, OA treatment could dramatically increase the intracellular ROS and O_2_^−^ levels with the enhancements of mean DCF and DHE fluorescence intensity, while the additional CACN could efficiently converse this phenomenon (Figures 1(c), 1(d), 1(g), and 1(h)), suggesting that CACN was capable of decreasing free radical generation under OA condition. Subsequently, another fluorescent probe, NDA, which was specific to glutathione (GSH), was applied to detect the intracellular GSH level (Figures 1(e) and 1(i)). Interestingly, OA indeed obviously reduced the basal intracellular GSH level, in comparison with controls. However, CACN, as an effective antioxidant, could attenuate OA-induced GSH depletion in a dose-dependent manner. Additionally, OA stimulation led to a mitochondrial membrane potential (MMP) motivation, inducing oxidative stress and mitochondria damage (Figures 1(f) and 1(j)). CACN treatment arrested the increase of MMP, showing a benefit effect against mitochondria dysfunction from OA-induced hepatocyte injury.

ACNs exhibit various excellent properties on preventing oxidative damage and various diseases, no matter in in vitro or in vivo studies, owing to their powerful ability of free radical clearing [31]. Cyanidin-3-rutinoside occupies the highest concentration in CACN, probably exerting the most key role in the cellular protective effect. And it has been reported to have enormous antioxidative activity, which might contribute to lipid-lowering both in vitro and in vivo trails [32, 33]. However, the lipid-lowering efficacy and mechanisms of cyanidin-3-rutinoside are still unclear; therefore, we next detected the benefit effect of CACN on OA-induced hepatic lipid accumulation.

### 3.3. CACN Improved Hepatic Lipid Accumulation

**Figure 2:**
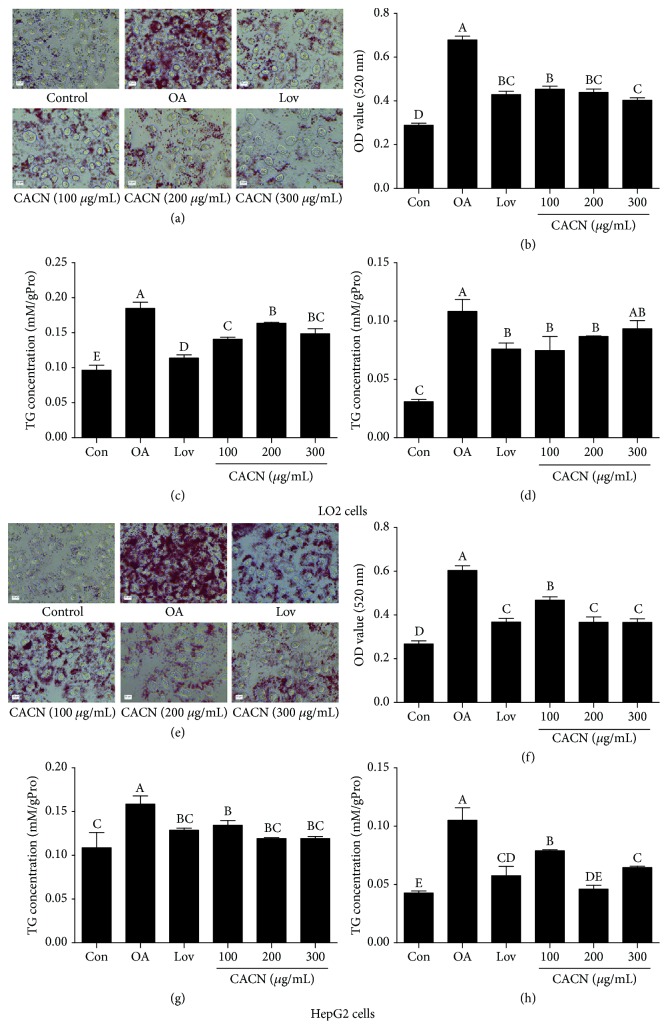
CACN promote lipid clearance in hepatic cells. (a) Representative images of oil red O-stained LO2 cells. (b) Quantitative results of oil red O staining in LO2 cells. (c) TC concentration in LO2 cells. (d) TG concentration in LO2 cells. (e) Representative images of oil red O-stained HepG2 cells. (f) Quantitative results of oil red O staining in HepG2 cells. (g) TC concentration in HepG2 cells. (h) TG concentration in HepG2 cells.

LO2 cells were treated with 100, 200, and 300 *μ*g/mL CACN in the presence of OA for 24 h to measure the inhibitory effect of CACN on OA-induced lipid overaccumulation. Lovastatin, a widely used lipid-lowering drug, was regarded as a positive control here [34]. As shown in Figure 2(a), compared with the model group, the number and size of intracellular lipid drops were significantly decreased after treatment with lovastatin and CACN, according to the results of oil red O staining using an optical microscope. Also, to quantify the cell lipid content, isopropanol was added to dissolve the intracellular oil red O, and the OD values at 520 nm (Figure 2(b)) showed that cell lipid droplets decreased simultaneously following CACN treatment in a dose-dependent manner. Moreover, quantification of intracellular TC and TG further indicated the overaccumulation of lipids in LO2 cells incubated with OA. And consistently, CACN blocked OA-mediated intracellular TG and TC content increase (Figures 2(c) and 2(d)). Meanwhile, HepG2, a widely used hepatocarcinoma cell strain, was also applied to further detect the mitigation effect on fat deposition in the hepatocytes. And CACN also decrease fat levels in HepG2 cells on the grounds of oil red O staining and TC and TG results (Figures 2(e)–2(h)). Taking all these data together, we confirmed that CACN show a significant protective effect on OA-induced lipid accumulation.

### 3.4. CACN-Induced Lipid Clearance Is along with a Coordinate Increase in Autophagy in Hepatic Cells

**Figure 3:**
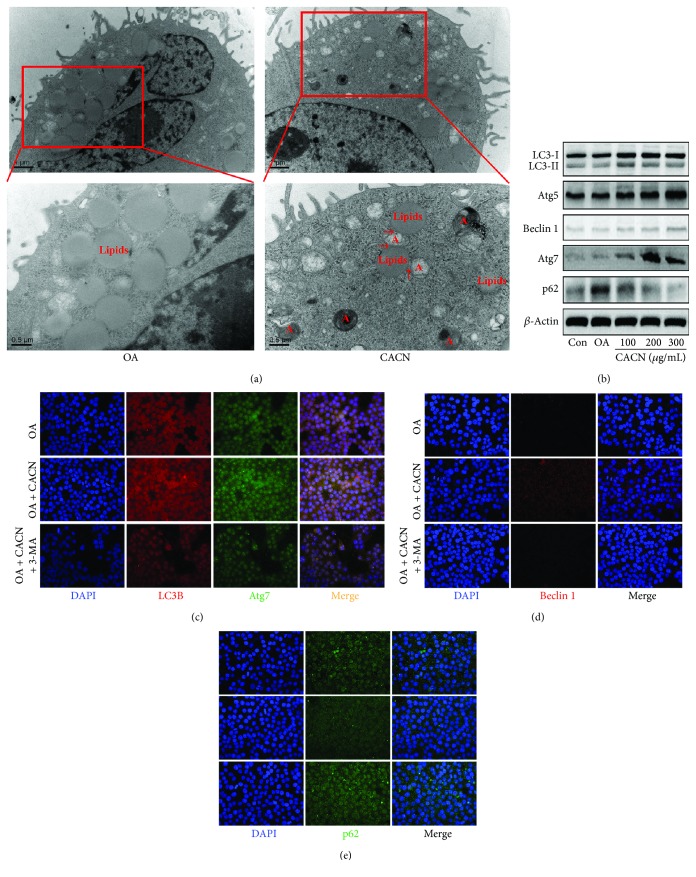
CACN activates autophagy in LO2 cells. (a) TEM images of LO2 cells (A means autophagy). (b) Effects of CACN on relative expressions of LC3II, Atg5, Atg7, Beclin1, and p62 in LO2 cells. (c) IF staining of LC3-II and Atg7 in LO2 cells. (d) IF staining of Beclin1 in LO2 cells. (e) IF staining of p62 in LO2 cells.

To gain insight into the relationship between CACN and NAFLD, we explored the potential mechanism for CACN induction of lipolysis in both LO2 and HepG2 cells. Previous studies reported that autophagy might be responsible for the clearance of excessive intracellular lipids in hepatocytes [35]. Thus, we focused on a newly found atypical lipolysis pathway: the autophagy pathway. We employed various methods to evaluate autophagic flux. Firstly, we explored whether the levels of autophagy in hepatic cells were affected after CACN exposure by detecting the number of autophagosomes in LO2 cells. As depicted in TEM images (Figure 3(a)), there are amounts of lipid droplets and almost no autophagosome under OA condition, while the CACN-treated group showed the decrease of the lipid droplet number and the increase of the autophagosome number. More interestingly, the colocalization of lipid droplets within the autophagosome compartment was observed (red arrows in Figure 3(a)), indicating the involvement of the autophagy pathway in reducing intracellular lipid by CACN. Secondly, expressions of the autophagy indicator LC3-II and other autophagy-related proteins (Atg5, Atg7, Beclin1, and p62) were detected by western blot assay in OA-stimulated LO2 cells. Compared with the control group, OA did not show a significant effect on LC3-II, Atg5, Atg7, and Beclin1 expression levels, while CACN significantly upregulated LC3-II, Atg5, Atg7, and Beclin1 and downregulated p62 expression levels compared with cells treated with OA solely at the same experimental conditions, suggesting that CACN indeed enhanced autophagic flux in LO2 cells (Figure 3(b)).

Furthermore, to further monitor autophagic fluctuation, LC3-II, Atg7, Bcelin1, and p62 levels were measured in the presence of CACN (200 *μ*g/mL) or 3-MA (a PI3K inhibitor, 10 mM) via immunofluorescent (IF) assay. As shown in Figures 3(c)–3(e), immunoblotting of LC3-II, Atg7 (Figure 3(c)), and Bcelin1 (Figure 3(d)) in CACN-treated cells showed a significant increase with the enhancement of fluorescence intensity, while p62 (Figure 3(e)) in CACN-treated cells showed a significant reduction, indicating increased autophagic flux. In addition, 3-MA challenge also resulted in the accumulation of LC3-II, Atg7 (Figure 3(c)), and Bcelin1 (Figure 3(d)) in OA-stimulated LO2 cells after incubation with CACN for 24 h compared with LO2 cells treated with OA alone, suggesting that CACN treatment promoted cellular autophagic processes even under 3-MA condition in OA-treated LO2 cells.

**Figure 4:**
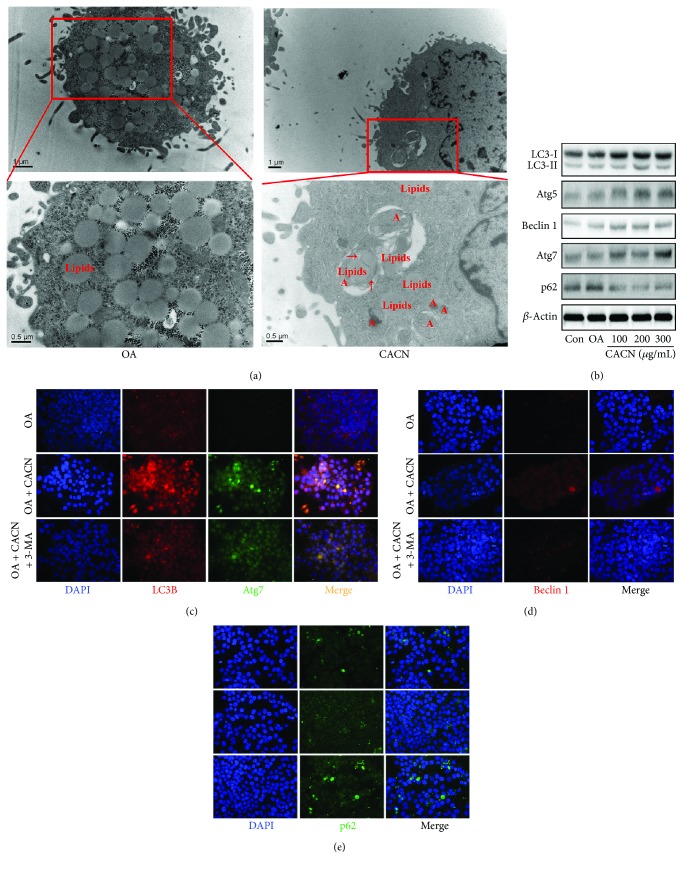
CACN activates autophagy in HepG2 cell cells. (a) TEM images of HepG2 cells (A means autophagy). (b) Effects of CACN on relative expressions of LC3II, Atg5, Atg7, Beclin1, and p62 in HepG2 cells. (c) IF staining of LC3-II and Atg7 in HepG2 cells. (d) IF staining of Beclin1 in HepG2 cells. (e) IF staining of p62 in HepG2 cells.

Additionally, the similar results were got in HepG2 cells after CACN treatments. TEM results showed the decrease of lipid droplets, increase of autophagosomes, and lipid droplets within the autophagosome compartment (Figure 4(a)). Western blot analysis indicated the upregulation of LC3-II, Atg5, Bcelin1, and Atg7 (Figure 4(b)), and the IF images clarified the enhancement of autophagic flux under OA and 3-MA conditions (Figures 4(c)–4(e)).

**Figure 5:**
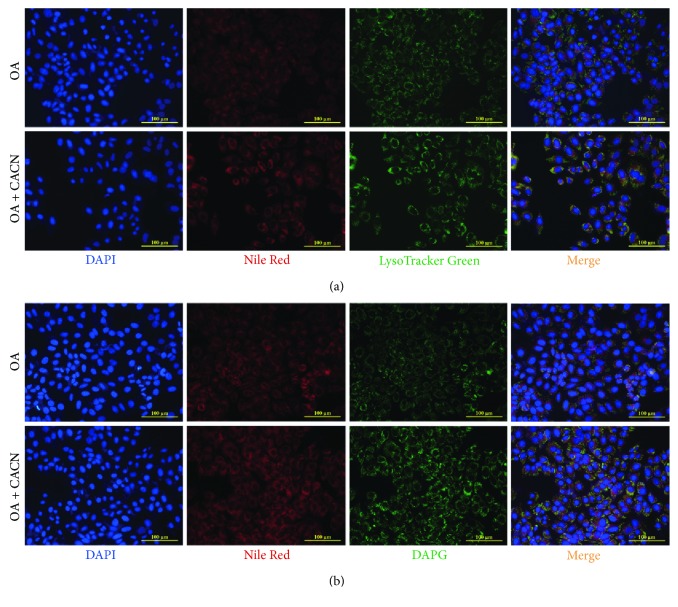
Colocalization of lysosomes or autophagosome with lipid droplets in HepG2 cells. (a) Double labeling of oil droplets (Nile Red) and lysosomes (LysoTracker Green) showing increased colocalization of lysosomes with cellular lipids (yellow dots) upon CACN treatment versus OA-alone-treated cells. (b) Double labeling of oil droplets (Nile Red) and autophagosomes (DAPG) showing increased colocalization of autophagosomes with cellular lipids (yellow dots) upon CACN treatment versus OA-alone-treated cells.

Finally, to confirm the autophagy induction effect of CACN, the colocalization of lysosomes or autophagosomes with lipid droplets was detected by double labeling of oil droplets (Nile Red) with lysosomes (LysoTracker Green) autophagosomes (DAPG) in HepG2 cells. As shown in Figure 5, the colocalization of lysosomes (Figure 5(a)) and autophagosomes (Figure 5(b)) with cellular lipids (yellow dots) upon CACN was markedly enhanced versus OA-alone-treated cells. Based on the former results, we confirmed that CACN possessed mighty proautophagic actions.

### 3.5. mTOR Pathways Were Involved in CACN-Induced Autophagy and Lipid Clearance

**Figure 6:**
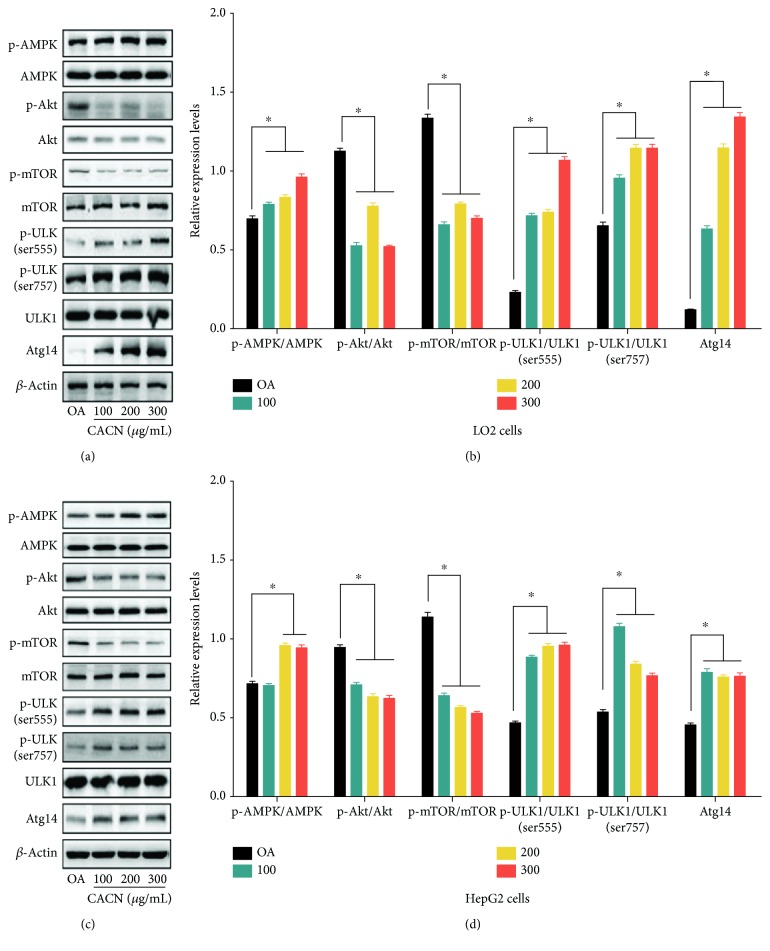
Effects of CACN on protein mTOR pathways in hepatic cells. (a) Bands of p-AMPK, AMPK, p-Akt, Akt, p-mTOR, mTOR, p-ULK1 (ser555), p-ULK1 (ser757), ULK1, and Atg14 of LO2 cells. (b) Relative expressions of p-AMPK/AMPK, p-Akt/Akt, p-mTOR/mTOR, p-ULK1 (ser555)/ULK1, p-ULK1 (ser757)/ULK1, and Atg14 in LO2 cells. (c) Bands of p-AMPK, AMPK, p-Akt, Akt, p-mTOR, mTOR, p-ULK1 (ser555), p-ULK1 (ser757), ULK1, and Atg14 of HepG2 cells. (d) Relative expressions of p-AMPK/AMPK, p-Akt/Akt, p-mTOR/mTOR, p-ULK1 (ser555)/ULK1, p-ULK1 (ser757)/ULK1, and Atg14 in HepG2 cells.

Our current study indicated that CACN could activate autophagy in hepatic cells accompanied by lipid clearance. Next, we investigated the underlying mechanisms of the process involved. The mammalian target of rapamycin (mTOR), as an inhibitor of autophagy and central sensor for energy states, growth factors, and nutritional signals, plays a central role in autophagy regulation [36]. It is reported that mTOR is positively regulated by the phosphorylation of Akt and negatively regulated by AMPK [37]. To study the possible involvement of AMPK, Akt, and mTOR in CACN-stimulated autophagy, we detected the effect of CACN on AMPK, Akt, and mTOR and the down signals (ULK1 and Atg14) using western blotting in both LO2 and HepG2 cells (Figure 6). The results depicted that phosphorylated AMPK was significantly upregulated by CACN, while pretreating cells with CACN alleviated OA-induced expression of phosphorylated Akt. Furthermore, we additionally measured the effect of OA on the mTOR pathway, which is widely served as the downstream target of AMPK and Akt. Finally, the phosphorylation (ser555 and ser757) of ULK1 and the relative expression of Atg14 were enhanced and benefit for autophagy induction. Our results illustrated that CACN significantly downregulated the phosphorylation of mTOR, suggesting that both the AMPK/mTOR signaling pathway and Akt/mTOR signaling pathway were involved in CACN-induced autophagy.

### 3.6. Autophagy Is Required for CACN-Induced Lipid Clearance

**Figure 7:**
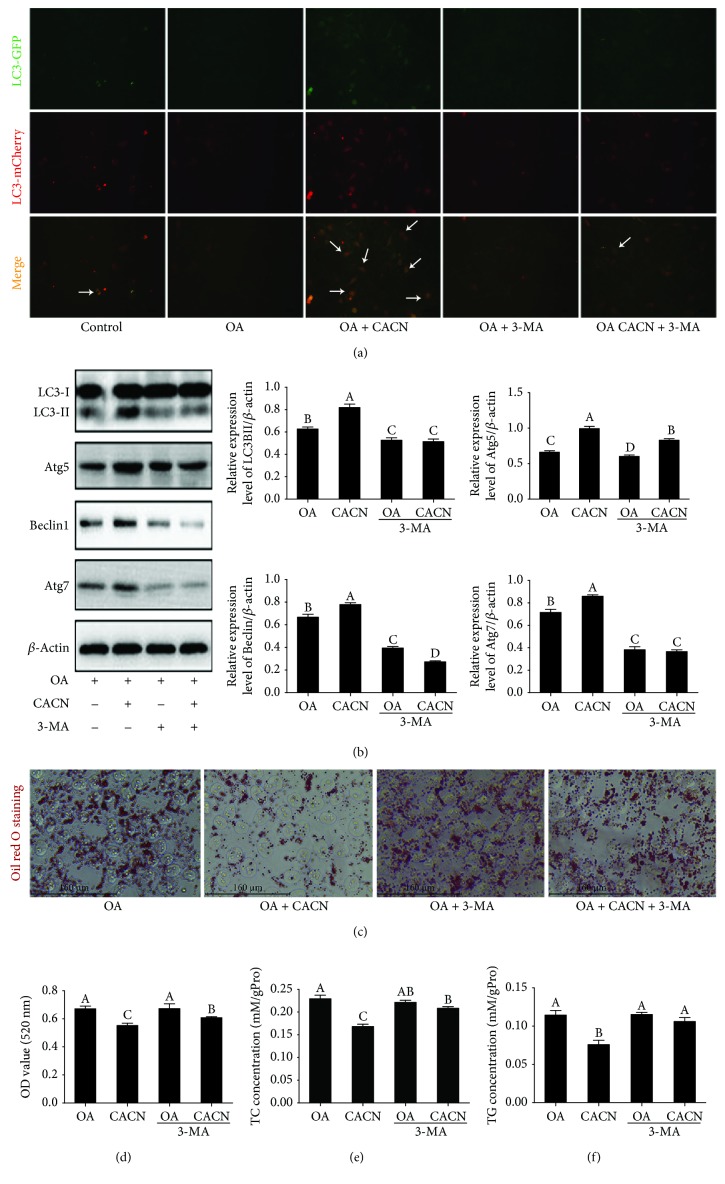
Autophagy is required for CACN-induced lipid clearance in LO2 cells. (a) Ad-mCherry-GFP-LC3B transfection (white arrows represent LC3II). (b) Effects of CACN on relative expressions of LC3II, Atg5, Atg7, and Beclin1 under 3-MA condition in OA-induced LO2 cells. (c) Representative images of oil red O-stained LO2 cells under 3-MA. (d) Quantitative results of oil red O staining under 3-MA condition in OA-induced LO2 cells. (e) TC concentration under 3-MA condition in OA-induced LO2 cells. (f) TG concentration under 3-MA condition in OA-induced LO2 cells.

**Figure 8:**
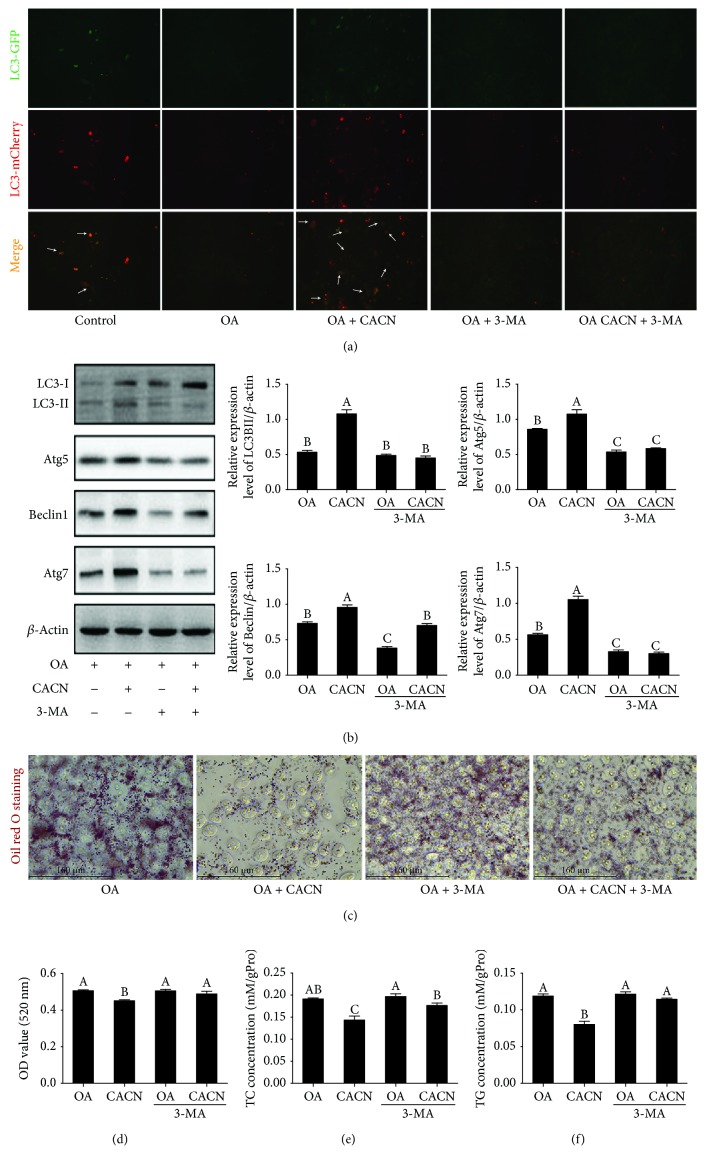
Autophagy is required for CACN-induced lipid clearance in HepG2 cells. (a) Ad-mCherry-GFP-LC3B transfection (white arrows represent LC3II). (b) Effects of CACN on relative expressions of LC3II, Atg5, Atg7, and Beclin1 under 3-MA condition in OA-induced HepG2 cells. (c) Representative images of oil red O-stained HepG2 cells under 3-MA. (d) Quantitative results of oil red O staining under 3-MA condition in OA-induced HepG2 cells. (e) TC concentration under 3-MA condition in OA-induced HepG2 cells. (f) TG concentration under 3-MA condition in OA-induced HepG2 cells.

Previous studies suggested that activation of autophagy protected hepatocytes from NAFLD both in vitro and in vivo [38, 39]. In order to detect whether CACN-inducedautophagy was involved in its protective effect on lipid accumulation, 3-MA was applied to inhibit autophagy and monitored their beneficial effect on lipid accumulation. First, ad-mCherry-GFP-LC3 staining and western blot analyses were applied to demonstrate autophagic flux in LO2 cells. In ad-mCherry-GFP-LC3 staining, GFP bonded to LC3 measures only autophagosomes, while mCherry detects both autophagosomes and lysosomes. The merge of green and red fluorescence shows yellow dots and indicates autophagosomes. The declined level of LC3II was observed in the OA and OA+3-MA groups compared with the control group. Conversely, the increased level of LC3II was shown in the OA+CACN and OA+CACN+3-MA groups, indicating that the OA-induced decrease in autophagy was prevented by 3-MA and heightened by CACN stimulation (Figure 7(a)), which corresponds to the IF results (Figures 3 and 4). Similarly, western blot data demonstrated that CACN could enhance LC3II, Atg5, Bclin1, and Atg7 relative expressions under OA condition, whereas this improvement of the autophagic flux of CACN was significantly weakened when exposed to 3-MA (Figure 7(b)). Interestingly, the intracellular lipid level in the 3-MA+CACN+OA group was higher than that in the CACN+OA group accompanied with the inhabitation of autophagy by 3-MA, according to the oil red O staining and TC and TG assays (Figures 7(c)–7(f)). These data together illustrated that autophagy was required for CACN-induced lipid clearance in LO2 cells, which also corresponds with the TEM results (Figures 3(a) and 4(a)). Additionally, the similar studies conducted with HepG2 cells show the similar conclusion (Figure 8), which further confirms that autophagy is involved in the antisteatosis benefits of CACN in hepatocytes.

**Figure 9:**
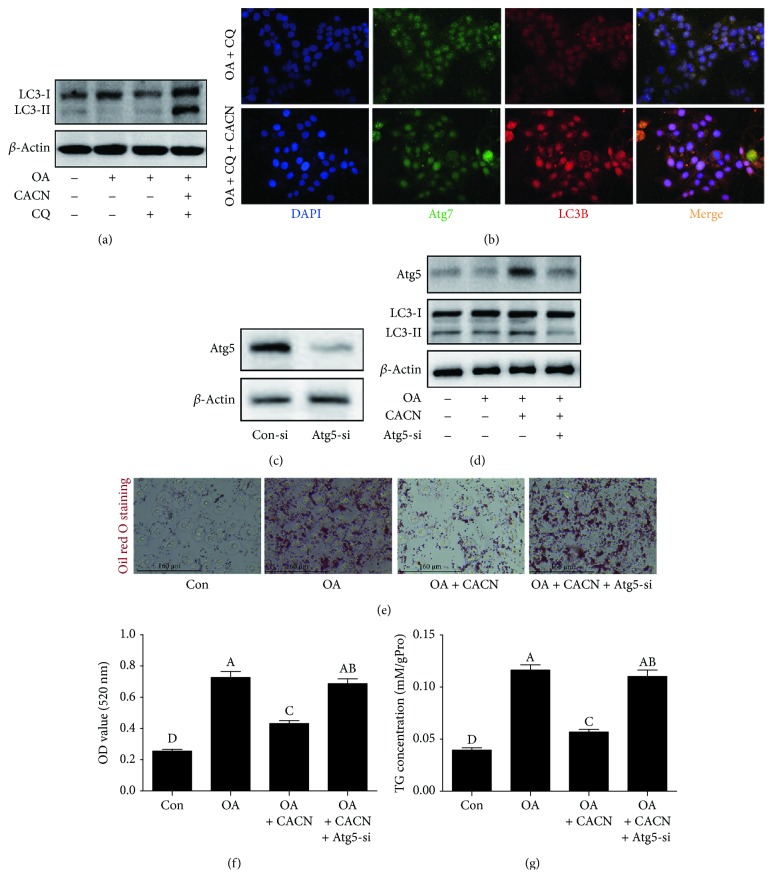
Autophagy is induced and essential for CACN-induced lipid clearance in HepG2 cells. (a) Relative expressions of LC3 under CQ condition in OA-induced HepG2 cells. (b) IF staining of LC3-II and Atg7 under CQ condition in OA-induced HepG2 cells. (c) Relative expressions of Atg5 under control-si and Atg5-si conditions in HepG2 cells. (d) Relative expressions of Atg5 and LC3 under Atg5-si condition in OA-induced HepG2 cells. (e) Oil red O staining under Atg5-si condition in OA-induced HepG2 cells. (f) Quantitative results of oil red O staining under Atg5-si condition in OA-induced HepG2 cells. (g) TG concentration under Atg5-si condition in OA-induced HepG2 cells.

To further determine whether or not autophagy is induced or blocked (downstream) under CACN conditions, a downstream autophagy inhibitor chloroquine (CQ) was applied. As depicted in Figures 9(a) and 9(b), CACN could also enhance autophagic flux under CQ condition, illustrating that the CACN-induced autophagic flux increase was through inducing autophagy, not blocking the degradation of autophagosomes in HepG2 cells. Also, considering that the efficacy and side effects of 3-MA were highly debated, Atg5-siRNA was applied to block autophagosome formation and analyzed its effect on the reduction of intracellular lipid to further confirm that autophagy induced by CACN is directly involved in reducing intracellular lipid content. We easily found that Atg5-siRNA not only reduced Atg5 and LC3 II expression levels (Figure 9(c)) but also notably decreased CACN-mediated reduction of intracellular lipid (Figures 9(d)–9(f)), strongly indicating the involvement of CACN-induced autophagy in the reduction of intracellular lipid.

## 4. Discussion

The liver, although not regarded as a primary lipid storage organ, plays a central role in lipid metabolism [40]. When lipid amasses, fats are mainly stored as TGs in hepatic cells, contributing to steatosis, which served as a main histologic feature of nonalcoholic fatty liver disease [41]. Although the therapeutic strategies of NAFLD are not fully clarified, studies have suggested that natural products play a key role in FFA-induced lipid accumulation [42]. Sweet cherry is a natural ACN-abundant plant and has been generally reported to attenuate impaired glucose and lipid homeostasis in FFA-induced lipid accumulation and high-fat diet-induced NAFLD mice [24, 25, 43, 44]. It is generally accepted to have beneficial effects on energy metabolism due to its role as a powerful antioxidant. In our previous study, we provided the evidence for the protective effect of CACN on high-fat diet-induced obesity and NAFLD, while the potential molecular mechanisms underlying the bioactivities have been understudied.

Autophagy is a well-regulated intracellular catabolic mechanism to maintain the cells' survival under stress which refers to self-eating [45]. Previous studies have reported that lipids were disintegrated via selective autophagy, known as lipophagy, and suggested that autophagy is an important composition in the regulation of lipid storage and lipid metabolism in the liver [46]. Researchers found that hepatocytes are exposed to 3-methyladenine, the pharmacological autophagy inhibitor, or treated with siRNA against the autophagy-related gene Atg5 to block autophagy, resulting in the excessive lipid accumulation with free fatty acid (FFA) treatments [9]. Also, Atg5 knockout mouse embryonic fibroblasts also displayed increased triglyceride levels [47]. These studies indicated that inhibition of autophagy led to lipid overaccumulation with FFA treatments and activation of autophagy acts conversely. Also, the accumulated data showed that autophagy activation protects hepatocytes against lipidoses [38], while the effect of ACN exposure on autophagy induction remains under studying and needs further attention.

Therefore, we asked ourselves whether CACN was capable of conferring their antilipidosis benefit via activating autophagy. We first measured the regulatory effect of CACN on OA-induced lipid accumulation and oxidative stress in human hepatocytes. We found that CACN was a powerful antioxidant with efficient lipid-lowering ability, which corresponds to previous studies [24, 25, 48]. Then, we explored the regulatory effect of CACN on autophagy in both LO2 and HepG2 cells. The results suggested that CACN is a mighty autophagy inducer evidenced by the findings that CACN exposure led to a significant increase of the number of autophagosome and the relative expression levels of LC3 II, Atg5, Beclin1, and Atg7. Subsequently, we mainly focused on the underlying connections between CACN-induced autophagy and lipid clearance under OA condition. The untreated control was discarded, which may not be optimal but should be sufficient to draw a conclusion that autophagy is required for CACN-induced lipid clearance. Western blot results implied that the AMPK/mTOR signaling pathway and Akt/mTOR signaling pathway were involved in CACN-induced autophagy against OA-induced lipid accumulation. Furthermore, 3-MA, a well-studied autophagy inhibitor, was engaged in to explore whether CACN-induced autophagy was essential to lipid clearance in hepatocytes. In line with our assumption, CACN-induced lipid clearance is along with a coordinate increase in autophagy in hepatic cells, and autophagy was required for this clearance. These results taken together indicate that CACN protects against OA-induced lipid accumulation via autophagy induction.

## 5. Conclusions

In summary, the present study demonstrated that CACN supplementation reduced oxidative stress and the levels of TC and TG in steatotic hepatic cells and autophagy induction via mTOR signaling pathways assisted in the lipid-lowering property. Our findings present a new mechanism that may contribute to understand the beneficial functions of CACN and illustrate that CACN is an effective therapy tactic of NAFLD and other metabolic disturbances. Although both LO2 and HepG2 lines were engaged, it is obvious that our result should be further strengthened with primary hepatocytes and in vivo studies to test the therapeutic potential of CACN in NAFLD.

## Data Availability

The data used to support the findings of this study are available from the corresponding author upon request.
